# Primary Bacterial Peritonitis in a Young Man: A Rare Manifestation of Invasive Group A Streptococcal Infection

**DOI:** 10.7759/cureus.73549

**Published:** 2024-11-12

**Authors:** Timour Gizzatullin

**Affiliations:** 1 Intensive Care Unit, Centre Hospitalier de Wallonie Picarde, Tournai, BEL

**Keywords:** critical care, epidemiology, invasive group a streptococcus infections, primary bacterial peritonitis, streptococcal toxic shock syndrome

## Abstract

Group A *Streptococcus* (GAS) is a ubiquitous pathogen responsible for a wide range of infections, from superficial to severe invasive forms (iGAS). Among these, primary bacterial peritonitis (PBP) due to GAS is a rare but severe presentation.

Recent epidemiological data indicate a significant rise in iGAS cases globally, which may be linked to changes in post-pandemic pathogen circulation.

This report describes a case of PBP and streptococcal toxic shock syndrome (STSS) caused by *Streptococcus pyogenes* in a young man with no known risk factors. To our knowledge, this is one of only 10 cases of PBP in men reported in the literature.

In this article, we review the epidemiology, risk factors, clinical manifestations, and management of iGAS, especially in the case of peritoneal involvement. Further research is needed to better understand the pathogenesis and optimize treatment strategies for this severe infection.

## Introduction

Group A *Streptococcus* (GAS) is a ubiquitous pathogen responsible for a wide range of infectious diseases. Renowned for its high virulence potential, GAS is frequently implicated in invasive infections, particularly affecting vulnerable patients. The clinical presentations of GAS infections are highly variable, ranging from superficial infections to toxic shock syndrome. In the years following the SARS-CoV-2 pandemic, a noticeable increase in invasive GAS infections has been observed worldwide [1].

In addition to typical presentations, such as skin and soft tissue infections, ear, nose, and throat (ENT) infections, and respiratory tract infections, peritoneal involvement is more rarely observed. Spontaneous peritonitis caused by *Streptococcus pyogenes* is an uncommon occurrence, accounting for only about 2% of invasive cases [2], with the majority of affected individuals being women [3].

This case report aims to describe a rare presentation of GAS primary bacterial peritonitis (PBP) in a male patient and review current management practices and outcomes in similar cases. Continued surveillance, research, and reporting are essential to enhance our understanding and improve outcomes for patients affected by this rare but serious condition.

## Case presentation

A 35-year-old man was admitted to the emergency department, referred by his primary care physician due to fatigue and dyspnea associated with profuse non-bloody diarrhea. Upon admission, he appeared pale, diaphoretic, and tachypneic.

In the medical history, he reported no specific complaints other than diarrhea and profound fatigue. The patient experienced subfebrile episodes (37.8°C-37.9°C) in the days preceding admission, without any chills. There were no symptomatic individuals in his close contacts.

The patient had no significant medical history, worked in an office, and had not recently traveled. He is the father of two school-aged children. There was no history of immunosuppression, autoimmune disease, or corticosteroid use.

During the clinical examination, the patient exhibited signs of distributive shock (pallor, mottling, cold extremities, and weak pulses). No lesions or skin rashes were found. His abdomen was soft and depressible, and he reported diffuse discomfort with deep palpation. Peristalsis was present.

His axillary temperature was 37.1°C. He had no altered level of consciousness, with a Glasgow Coma Scale (GCS) score of 15. He was tachycardic at 140 bpm, maintaining a blood pressure of 92/48 mmHg, and was tachypneic with a respiratory rate of 26/minute and a pulse oxygen saturation (SpO2) of 95% on room air.

The initial blood gas analysis (Table 1) revealed compensated metabolic acidosis with a pH of 7.41, a low bicarbonate level of 12 mM, a pCO2 of 20 mmHg, and an elevated lactate level of 5.7 mM.

**Table 1:** Admission laboratory results, as well as results on day 1, day 2, week 1, and week 2 ALT: alanine aminotransferase, APTT: activated partial thromboplastin time, AST: aspartate aminotransferase, GGT: gamma-glutamyl transferase, LDH: lactate dehydrogenase, NLR: neutrophil-to-lymphocyte ratio, PaCO2: partial arterial pressure in carbon dioxide, PaO2: partial arterial pressure in oxygen The (+) and (-) signs indicate that the value deviates from the normal range.

Parameter	Day 0	Day 1	Day 2	Day 7	Day 14	Normal values
Hemoglobin (g/dL)	18.4 (+)	11.3	11.1	9.6	7.9	13.3-16.7
Hematocrit (%)	50 (+)	31	32	28 (-)	24 (-)	38-48
Leucocytes (/μL)	20.5×10^3^ (+)	22.6×10^3^ (+)	35.6×10^3^ (+)	27.1×10^3^ (+)	15.6×10^3^ (+)	4-10×10^3^
Neutrophiles (/μL)	17.3×10^3^ (+)	20.4×10^3^ (+)	33.1×10^3^ (+)	21.6×10^3^ (+)	12×10^3^ (+)	1.6-7×10^3^
Lymphocytes (/μL)	1.6×10^3^	0.5×10^3^	0.85×10^3^	1.6×10^3^	1.7×10^3^	0.8-5×10^3^
NLR	11.1 (+)	-	-	-	-	0.78-3.53
Platelets (/μL)	322×10^3^	169×10^3^	196×10^3^	502×10^3^ (+)	931×10^3^ (+)	150-450×10^3^
APTT (seconds)	35.3	-	-	-	-	25.1-36.5
Prothrombin time (seconds)	12.9	-	-	-	-	9.35-14.30
Thrombin time (seconds)	11.2	-	-	-	-	10-18
C-reactive protein (mg/L)	331.2 (+)	191.3 (+)	133.5 (+)	62.5 (+)	129.5 (+)	<5
Urea nitrogen (mg/dL)	137 (+)	178 (+)	193 (+)	139 (+)	17	15-50
Creatinine (mg/dL )	5.5 (+)	3.6 (+)	3.5 (+)	1.6 (+)	1	0.6-1.3
Total bilirubin (mg/dL )	2.9 (+)	1.1 (+)	0.8	1.6 (+)	0.9	<1.3
Direct bilirubin (mg/dL)	2.2 (+)	-	-	-	-	<0.4
Creatine kinase (U/L)	1297 (+)	1204 (+)	951 (+)	94	65	20-200
LDH (UI/L)	817 (+)	640 (+)	564 (+)	354 (+)	248	<250
AST (U/L)	327 (+)	355 (+)	150 (+)	64 (+)	53 (+)	15-40
ALT (U/L)	116 (+)	118 (+)	66 (+)	32 (+)	50 (+)	10-40
GGT (U/L)	55	19	16	189 (+)	103 (+)	55
pH	7.41	-	-	-	-	7.35-7.45
PaCO_2_ (mmHg)	20 (-)	-	-	-	-	35-45
PaO_2_ (mmHg)	81	-	-	-	-	85-95
HCO_3_^-^ (mmol/L)	12 (-)	-	-	-	-	22-28
Base excess (mmol/L)	-9.5 (-)	-	-	-	-	-2-2
Lactic acid (mmol/L)	5.7 (-)	-	-	-	-	0.5-2.2

Laboratory findings (Table 1) showed elevated hemoglobin and hematocrit levels, as well as ionic imbalances (low sodium, chloride, potassium, and bicarbonate levels), indicative of gastrointestinal losses. There was also a significant inflammatory response, acute kidney failure, and increased levels of creatinine phosphokinase (CPK), lactate dehydrogenase (LDH), aspartate transaminase (AST), and alanine transaminase (ALT).

**Figure 1:**
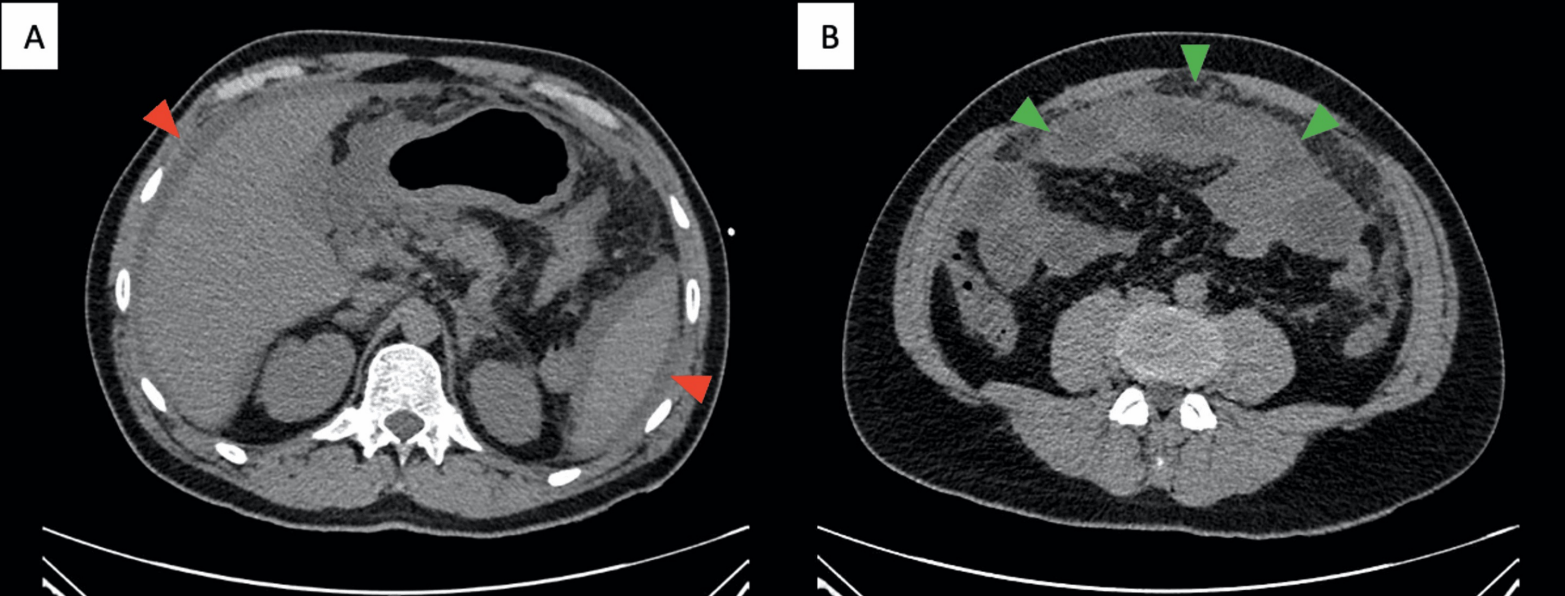
Computed tomography in spontaneous contrast on the day of admission Two images showing axial scans through the upper third (A) and middle third (B) of the abdomen. It shows diffuse perihepatic and perisplenic ascites (red arrows), diffuse mesenteric infiltration, and dilated small intestine loops (green arrows).

Given the clinical condition and abnormal laboratory results, an abdominal computed tomography (CT) scan without contrast was performed, revealing splenomegaly and diffuse infiltration of the mesentery and greater omentum, along with ascites, consistent with peritonitis (Figure 1).

The patient was started on antibiotic therapy with cefuroxime and metronidazole after obtaining bacterial cultures (urine, blood, and sputum). He was admitted to the intensive care unit (ICU). Several hours after admission, the clinical picture was complicated by deteriorating neurological status, requiring orotracheal intubation.

**Figure 2:**
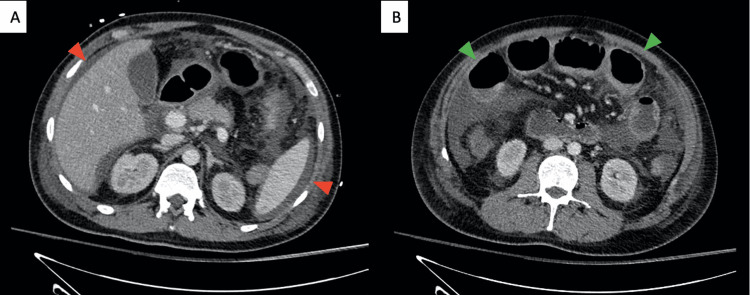
Contrast-enhanced computed tomography in the portal phase the day after admission Two CT images showing axial scans through the upper third (A) and middle third (B) of the abdomen. It shows multi-compartmental ascites (red arrows), peritoneal enhancement, dilated small intestine loops (green arrows), and no evidence of gastrointestinal perforation. CT: computed tomography

The assessment was further supplemented by a peritoneal tap, yielding purulent fluid, and an injected abdominal CT scan confirming peritoneal enhancement, increased multi-compartmental ascites, and splenomegaly without signs of intestinal perforation (Figure 2).

An exploratory laparoscopy was scheduled to search for possible gastrointestinal perforations and manage local sepsis. However, the procedure was quickly converted to laparotomy due to the inflammatory state, a significant amount of pus (1.5 L aspirated), and the presence of numerous false membranes and adhesions. The abdominal cavity exploration revealed purulent peritonitis in all four quadrants. The small intestine and colon were thoroughly examined, showing no signs of ischemia or perforation. The appendix and gallbladder were healthy. The stomach and duodenum had no perforations but exhibited a thickened area on the lesser curvature associated with the presence of fibrin and a larger quantity of false membranes. Samples were sent for bacteriological analysis, and the abdominal cavity was thoroughly irrigated before closure.

Bacteriological analyses of the peritoneal tap and intraoperative samples returned positive for *Streptococcus pyogenes*. This enabled the timely adjustment of the antibiotic therapy to a combination of amoxicillin-clavulanate and clindamycin, as recommended in invasive GAS infections. Furthermore, all other microbiological samples (blood and urine cultures) remained negative.

The patient's clinical course in the days following abdominal cavity cleaning was favorable, with resolution of the inflammatory syndrome and improved renal function. He was extubated on day 4 and left the intensive care unit on day 7.

## Discussion

Group A *Streptococcus* (GAS) is a ubiquitous pathogen responsible for a wide range of infectious diseases, including superficial infections as well as invasive forms (iGAS) that contribute significantly to global morbidity and mortality [4]. A large epidemiological study across 11 European countries estimated the annual incidence of iGAS at 2.79 per 100,000 inhabitants [5].

The high pathogenic potential of GAS is primarily due to several key virulence factors. Among these, the M protein stands out for its considerable antigenic variability (over 200 variants), making it a crucial tool for phenotyping different GAS strains. This protein also exerts immunomodulatory effects and shares similarities with host proteins, potentially leading to autoimmune disorders [4]. Protein S is another significant virulence factor that aids in the adhesion and lysis of erythrocytes and contributes to the suppression of the host’s immune response [6]. Additionally, the hyaluronic acid capsule promotes adhesion to epithelial cells and facilitates invasion through mucosal barriers [6].

Furthermore, GAS secretes a variety of exotoxins, including streptokinase, a protein without intrinsic proteolytic activity but with a potent ability to activate plasminogen [7]. Another key exotoxin is streptolysin O (SLO), which contributes to the lysis of cells and tissues. Variations in SLO expression and activity across different GAS strains may explain the diverse clinical manifestations observed in infected hosts [8]. Most notably, GAS produces superantigens, a family of exotoxins that bind to major histocompatibility complex (MHC) class II molecules outside of the typical antigen presentation sites and to T-cell receptors in a non-specific manner. This interaction forces abnormal connections between antigen-presenting cells and T lymphocytes, leading to disproportionate T-cell recruitment (up to 20%-30%) and a massive release of pro-inflammatory cytokines, as seen in streptococcal toxic shock syndrome (STSS) [9].

In the last quarter of 2022, there was a marked increase in iGAS cases in several European countries [1,10-12], as well as in North America [13] and Oceania [14]. This rise is likely related to the extensive recirculation of the pathogen following the easing of distancing measures implemented during the pandemic. The spread was greatly facilitated by a large pediatric vector that had not yet been exposed to GAS.

Belgium followed this trend, with a stable incidence of iGAS until 2021, which then increased from late 2022, reaching particularly high levels in 2023 [15]. The groups particularly affected remain young children under five years and adults over 65 years, with annual incidences of 25 and 14 cases per 100,000 inhabitants, respectively. The incidence in adults aged 36-65 years is approximately 6.5 cases per 100,000 inhabitants [15].

Risk factors predisposing to invasive forms in adults include tobacco, alcohol, or injectable drug use, diabetes, cardiovascular diseases, obesity, immunosuppression (e.g., immunosuppressive therapy, HIV infection, and active cancer), skin lesions, and a precarious socioeconomic status [2,5,16,17]. In the group of young adults (18-45 years), exposure to children with pharyngitis is another major risk factor, increasing the likelihood of developing iGAS disease (relative risk (RR): 4.93) [17].

Disease expression is highly variable and can affect all systems. However, the predominant invasive presentations among adults are soft tissue infections, pneumonia, and STSS [2,12,16-18]. Intra-abdominal infections account for only about 2% of presentations [2,16,17], primarily affecting individuals aged 40-59 years [16].

The morbidity and mortality associated with iGAS are high. Retrospective studies show high intensive care unit (ICU) admission rates ranging from 20% to 30% [10,19], with rates as high as 43% [16]. Surgical intervention is frequent, with rates of up to 31.9% [16] and 41% for the most severe patients [12]. In-hospital mortality is also very high, ranging from 10% to 20% in various cohorts [10,12,16,18] and up to 27% in some cases [19]. A Swedish study on ICU admissions between 2007 and 2019 reports particularly low ICU mortality rates (5.7%) with a median length of stay of only 3.8 days. However, the 180-day mortality rate increases to 23%, similar to the values found in previous cohorts [20].

The genotype primarily found in invasive forms is emm1 [2,10,12,16,18,20]. Work by Rodriguez-Ruiz et al. in 2023 reveals that during the period relevant to this clinical case, the M1UK lineage was predominant among emm1 clones. This form is not associated with more severe infection forms but seems to have a selection advantage over other lineages and more frequently contains the mefE gene, which encodes an efflux pump conferring resistance to macrolides [21].

In this clinical case review, the patient presents a severe form of iGAS. Upon admission, he showed signs of shock associated with acute renal failure and hepatic cytolysis. This presentation is consistent with the definition of STSS [22]. It represents a fulminant clinical presentation with rapidly evolving multisystem dysfunction complicating iGAS.

Pathogenesis is closely linked to the release of bacterial toxins that directly bind major histocompatibility complex class II to host CD4 T-cell receptors, bypassing the classical antigen presentation system. This interaction induces a cataclysmic activation of T lymphocytes and antigen-presenting cells with a massive release of inflammatory cytokines [9].

STSS most commonly complicates skin infections (necrotizing fasciitis and cellulitis) [5,9] and is mainly associated with streptococcal serotypes M1 and M3 [9,23]. According to cohorts, it affects between 4% and 13% of patients hospitalized for iGAS [2,5,16,17], but this rate can rise to 55% [12] and even 77% [18] in ICU patients, with significant mortality rates (26%-44%) [2,5,12,18].

Treatment relies on hemodynamic support and management of organ dysfunctions, surgical control of the infectious focus, and antimicrobial and immunomodulatory therapy.

In the presented clinical case, the patient initially received antibiotic coverage for intra-abdominal sepsis with second-generation cephalosporin and metronidazole. Following the identification of iGAS, antibiotic therapy was adjusted to a combination of amoxicillin-clavulanate and clindamycin.

The use of a combination of beta-lactam and clindamycin is recommended in invasive GAS infections, particularly in cases meeting STSS criteria [24,25]. Clindamycin, a lincosamide with bacteriostatic activity, blocks protein synthesis by binding to the 50S ribosomal subunit of bacterial ribosomes, thus inhibiting exotoxin production. Consequently, its activity is independent of the treatment duration [19] and bacterial inoculum [26]. Its addition reduces in-hospital mortality [19,27] with benefits that appear to persist regardless of the timing of treatment initiation and disease severity [19].

GAS susceptibility to penicillin remains 100%. However, an increase in variants with mutations in the penicillin-binding protein (PBP) reducing its affinity for beta-lactams has been observed, although not significantly [23]. In contrast to beta-lactams, the increasing rate of clindamycin-resistant strains is becoming a concern [23,26]. In Belgium, resistant strains accounted for 4.4% of samples in 2018 [15,16]. However, this rate is likely higher among socially disadvantaged populations, those without fixed addresses, or those living in communal settings [28].

Immunomodulatory treatment with intravenous immunoglobulin (IVIG) remains an option in treating patients with STSS. Experimental data show that IVIG can neutralize exotoxins and facilitate bacterial opsonization in addition to having a direct anti-inflammatory effect [29]. Its use has shown a mortality benefit in several prospective studies [30,31] and a meta-analysis [32]. However, due to the limited power and heterogeneity of these studies, no clear recommendations can be made regarding its use in STSS patients [24].

Although there is no consensus on the management of GAS primary bacterial peritonitis, as shown in Table 2, the vast majority of cases reported in the literature benefit from surgical exploration [3,33-44]. The goal is to identify and treat any potential source of infection, reduce the bacterial inoculum, and obtain microbiological samples. Laparoscopy can be preferred depending on material availability and surgical expertise. However, laparotomy is often necessary due to the difficulty of working in an inflamed abdomen and the non-negligible risk of hemodynamic instability secondary to pneumoperitoneum.

**Table 2:** Summary of clinical cases of GAS primary bacterial peritonitis reported since 1982 GAS: Group A *Streptococcus*, STSS: streptococcal toxic shock syndrome

Studies	Gender	STSS	Surgery	Local sepsis control
Iitaka et al. (2017) [3] and Westwood and Roberts (2013) [33]	Female: 39	Yes: 28	Yes: 40	Laparotomy: 30
	Male: 7	No: 17	No: 5	Laparoscopy: 10
	-	Unknown: 1	Unknown: 1	Percutaneous drainage: 1
Thomas et al. (2009) [34]	Female	Yes	No	Percutaneous drainage
Thomas et al. (2009) [34]	Male	Yes	Yes	Laparotomy
Iitaka et al. (2017) [3]	Female	No	No	-
Varela et al. (2018) [35]	Female	Yes	Yes	Laparoscopy
Wahab et al. (2018) [36]	Female	Yes	No	Percutaneous drainage
Ledger (2018) [37]	Female	No	No	-
Johnson et al. (2019) [38]	Male	Yes	Yes	Laparoscopy
Johnson et al. (2019) [38]	Female	No	Yes	Laparoscopy
Haskett et al. (2020) [39]	Female	No	No	Percutaneous drainage
Aw et al. (2021) [40]	Female	No	Yes	Laparoscopy
Holtestaul et al. (2021) [41]	Female	No	Yes	Laparoscopy
Sumiyama et al. (2022) [42]	Female	Yes	Yes	Laparotomy
Soga et al. (2023) [43]	Female	No	No	-
Matsumoto et al. (2024) [44]	Female	Yes	No	Percutaneous drainage
Total	Female: 51	Yes: 35	Yes: 47	Laparotomy: 32
	Male: 9	No: 24	No: 12	Laparoscopy: 15
		Unknown: 1	Unknown: 1	Percutaneous drainage: 5

In recent years, there has been a trend toward managing patients without resorting to surgery, even among the most severe cases with STSS. In such instances, treatment with antibiotics alone or in combination with percutaneous peritoneal lavage has been proposed [34,36,39,44]. Although these approaches are still not well-documented and definitive conclusions are lacking, the observed results seem encouraging. The choice between these strategies is typically guided by clinical presentation, severity of infection, and patient stability, with more severe cases generally necessitating surgery to promptly address the source of infection. In the case described above, the patient had a severe infection with multiple organ dysfunctions and a rapidly deteriorating condition, which justified our management approach.

Primary bacterial peritonitis (PBP) due to *Streptococcus pyogenes* is a rare manifestation of iGAS infection. Pathogenesis remains debated, and the portal of entry of the pathogen into the peritoneum is often unknown. However, as evidenced by the high predominance of females in documented cases, retrograde contamination from the urogenital system is strongly suspected. Hematogenous dissemination originating from ear-nose-throat or skin infections is also plausible. Our patient did not present any known risk factors for developing iGAS disease, except for having children of school age, who are known to be at higher risk for such infections [17].

In total, 60 adult cases have been described in the literature, with a very high proportion of affected women (85%). More than half of these cases are associated with STSS (59.3%), and a large majority have undergone surgical intervention (79.6%) (Table 2).

To our knowledge, only nine documented cases of primary GAS peritonitis concern men. Table 3 summarizes the nine cases described in the literature [34,38,45-51]. These involve young men aged between 36 and 44 years and a 14-year-old boy. Risk factors for invasive GAS infection are identified in only three of them. Most presented STSS criteria. Four patients were treated with an antibiotic in combination with clindamycin, two received IVIG treatment, and all underwent surgical management, seven of whom had open surgery. Although one patient required limb amputation, all were able to leave the ICU and return home.

**Table 3:** Summary of clinical cases of GAS primary bacterial peritonitis reported in men GAS: Group A *Streptococcus*, NSAID: non-steroidal anti-inflammatory drug, PID: pelvic inflammatory disease, STSS: streptococcal toxic shock syndrome, IVIG: intravenous immunoglobulin

Studies	Gender	Age	Risk factors	Primary source of infection	Blood cultures	STSS	Antibiotics after GAS identification	Surgery	Outcome
Gribbin and Cox (1990) [46]	Male	44	Alcohol abuse, smoker	-	Positive	No	Cephalothin	Laparotomy	Alive
Gelshorn et al. (1994) [47]	Male	46	-	Skin lesion	Positive	Yes	-	Laparotomy	-
Sanchez and Lancaster (2001) [48]	Male	34	-	-	Negative	No	Cephalexin + clindamycin	Laparoscopy	Alive
Gavala et al. (2002) [49]	Male	40	-	Pharyngitis	Negative	Yes	Penicillin over one year	Laparotomy	Alive
Kanetake et al. (2004) [50]	Male	40	Child with GAS pharyngitis	-	-	Yes	Fosfomycin + imipenem/cilastatin switched to cefoperazone/sulbactam + amikacin + IVIG	Laparotomy	Alive
Kinsella et al. (2009) [51]	Male	38	-	Skin lesion	Positive	No	Unknown	Laparotomy	Alive
Thomas et al. (2009) [34]	Male	36	NSAID previous month	-	Positive	Yes	Amoxicillin + gentamicin + clindamycin	Laparotomy	Alive
Monneuse et al. (2010) [45]	Male	-	-	-	-	-	Clindamycin + unknown	Laparotomy	Alive
Johnson et al. (2019) [38]	Male	14	-	-	-	Yes	Ertapenem followed by levofloxacin + clindamycin + IVIG	Laparoscopy	Alive

## Conclusions

The incidence of invasive Group A *Streptococcus* infections has significantly increased in recent years, with a notable rise observed across Europe. Among the various manifestations of iGAS, primary bacterial peritonitis (PBP) caused by* Streptococcus pyogenes* is exceedingly rare, particularly in men without significant predisposing factors.

The diagnosis of PBP is typically established through microbiological analysis of peritoneal fluid. Effective management primarily involves antibiotic therapy, often combining beta-lactams with clindamycin, and surgical intervention to control the infection source and reduce bacterial load. However, emerging conservative treatment strategies, including antibiotic therapy alone or combined with percutaneous peritoneal lavage, may offer alternative options for patient management.
